# Predictive deep learning model based on contrast-enhanced mammography for breast cancer diagnosis: a pilot study

**DOI:** 10.1093/bjrai/ubag013

**Published:** 2026-06-29

**Authors:** Roberto Maroncelli, Chiara De Nardo, Veronica Rizzo, Federica Cicciarelli, Marcella Pasculli, Francesca Galati, Federica Pediconi

**Affiliations:** Department of Radiological, Oncological and Pathological Sciences, Sapienza—University of Rome, 00185 Rome, Italy; Department of Experimental Medicine, Sapienza—University of Rome, 00161 Rome, Italy; Department of Radiological, Oncological and Pathological Sciences, Sapienza—University of Rome, 00185 Rome, Italy; Department of Radiological, Oncological and Pathological Sciences, Sapienza—University of Rome, 00185 Rome, Italy; Department of Radiological, Oncological and Pathological Sciences, Sapienza—University of Rome, 00185 Rome, Italy; Department of Radiological, Oncological and Pathological Sciences, Sapienza—University of Rome, 00185 Rome, Italy; Department of Radiological, Oncological and Pathological Sciences, Sapienza—University of Rome, 00185 Rome, Italy; Department of Radiological, Oncological and Pathological Sciences, Sapienza—University of Rome, 00185 Rome, Italy

**Keywords:** contrast enhanced mammography, deep learning, artificial intelligence, digital breast tomosynthesis, breast cancer

## Abstract

**Objectives:**

The interpretation of contrast-enhanced mammography (CEM) images heavily depends on radiologists’ expertise, highlighting the need for automated tools to assist clinical decision-making. This pilot study aimed to develop a deep learning model using CEM images to predict the histological diagnosis of breast cancer.

**Methods:**

We retrospectively analyzed patients who underwent CEM followed by histopathological assessment (October 2022-May 2023) across 2 centers. CEM images from center 1 were used for model development, including training and 10-fold cross-validation, while images from center 2 served as an independent external test set. The breast region was manually segmented, and 2 deep learning architectures were implemented as ensemble classifiers, using biopsy results as reference standard. Performance metrics included accuracy, sensitivity, specificity, positive predictive value (PPV), negative predictive value (NPV), and area under the Receiver Operating Characteristic Curve (ROC) curve (AUC).

**Results:**

A total of 106 CEM images were retrospectively analyzed (50 from center 1 and 56 from center 2), obtained using different mammography systems. Histopathology identified 51 lesions (48%) as malignant and 55 (52%) as benign. Cross-validation yielded an ROC-AUC of 75% [58.4-91.6], accuracy of 68.7% [58.3-79], sensitivity of 68.9% [52.2-85.6], specificity of 67.8% [65.4-70.2], PPV of 69.3% [55.5-83.1], and NPV of 73.5% [55.2-91.8] (*P* < .05). External testing on images from center 2 (27 malignant, 29 benign) achieved an accuracy of 64.3%.

**Conclusions:**

The study showed promising performance but requires further development and dataset expansion. The model has the potential to be integrated into clinical practice.

**Advances in knowledge:**

A deep learning model based on CEM provides a tool to support physicians in the decision-making process and reduce invasiveness in breast cancer management.

## Graphical Abstract

**Figure:**
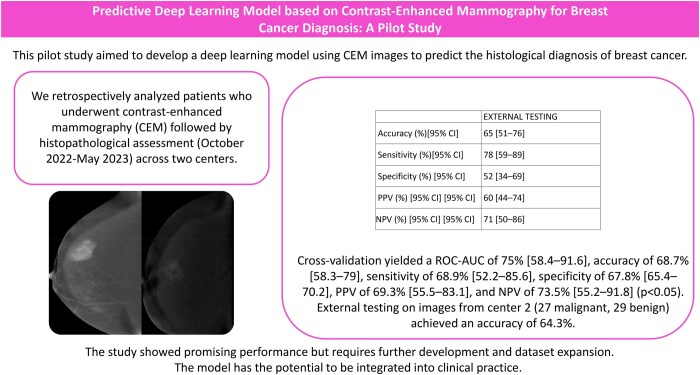

## Introduction

Breast cancer is the most common cancer among women worldwide.^1^

According to the World Health Organization (WHO), there were approximately 2.3 million new cases of breast cancer globally in 2022, making it the most prevalent cancer in women.^2^ Early detection is crucial for improved prognosis.^3^

Breast screening is an effective method for detecting early-stage cancer and has been shown to improve survival rates significantly.^4^

Digital mammography (DM) and digital breast tomosynthesis (DBT) detect structural changes in breast tissue, opacity, architectural distortions or microcalcifications.^5^

While both methods are crucial for breast cancer screening, particularly in asymptomatic patients, their effectiveness can be limited in women with dense breast tissue.^6^

In such cases, distinguishing lesions becomes more challenging due to the density of the surrounding tissue.^7^

Contrast-enhanced mammography (CEM) represents a promising emerging technique in breast imaging.^8^

As a hybrid imaging modality, CEM combines the morphological characteristics of conventional mammography with functional information obtained from intravenous iodinated contrast agents.^9^

This dual functionality allows for a detailed assessment of both lesion morphology and tumor angiogenesis, providing superior performance compared to traditional methods such as DM, ultrasound (US), and digital breast tomosynthesis (DBT).^10^

CEM uses a dual-energy technique to produce high-resolution, low-energy digital mammogram images. These images are then combined to create a digitally subtracted image, which can be useful for identifying the vascularity of a specific lesion.

CEM generates 3 types of images for both the craniocaudal (CC) and mediolateral oblique (MLO) views: high-energy (HE), low-energy (LE), and dual-energy subtraction (DES) images.^11^

However, like all imaging techniques, the assessment of CEM images relies on the radiologist’s expertise and experience; this creates a significant challenge in developing automated or semi-automated techniques that provide decision support.^12^

Recent major advancements in this field are driven by the use of artificial intelligence to process vast amounts of data from various imaging modalities.^13^

Radiomics is the process of extracting quantitative characteristics, known as features, from medical images.^14^ Feature extraction typically involves the use of pattern recognition algorithms to generate a set of numerical values. Each of these values quantitatively represents a specific geometrical or physical property of the selected portion of an image. In the context of tumor characterization, the radiomic features most analyzed are those that describe the tumor’s size, shape, intensity, and texture.^15^ This study aimed to develop and validate a deep learning model utilizing (CEM) images to distinguish between malignant and benign lesions, with histopathological examination serving as the gold standard for comparison.

## Methods

### Study design and patient population

This bicentric, retrospective study was approved by the Institutional Review Boards (IRBs) of both participating centers, with informed consent requirements waived. All patient data was handled in accordance with relevant data protection regulations and IRB guidelines.

This study was designed, conducted, and reported in adherence to the Checklist for Clear Reporting of Radiomics Studies (CLEAR) to ensure methodological rigor and transparency.

We retrospectively selected patients from 2 hospitals (Center 1 and Center 2) who underwent CEM between October 2022 and May 2023.

The patients included in the study had undergone CEM as a second-level examination due to a suspicious lesion detected during standard mammography and they subsequently underwent a biopsy.

Subsequently, to evaluate and compare the performance of the radiomics-based model with the radiologist’s assessment, additional patients who underwent CEM at a different hospital and with a different mammography system were included in the study.

The collected cases were used exclusively for the development and validation of the radiomics model.

This approach ensured a separation between the cases used for model development and validation and those used for comparison, maintaining methodological integrity.

Inclusion criteria: patients with suspicious breast lesions histologically proven, and that underwent dual-energy CEM.

Exclusion criteria: patient with breast implants, presence of nonremovable drilling at the nipple, pacemakers, clips or other metal implants, pregnancy or possible pregnancy, inability to keep upright immobility during the examination, renal disease, or chemotherapy treatment at the time of imaging.

As a pilot study, the sample size was determined based on the availability of high-quality CEM data with complete histological confirmation across the participating centers during the study period. The strict selection criteria focused on patients undergoing CEM as a second-level examination for suspicious lesions. This specific population was chosen to test the model’s performance in a challenging diagnostic setting where automated decision support could provide the most clinical value, rather than in a general screening population.

No missing data were present in the study dataset. All cases included had complete imaging, clinical, and histopathological data, ensuring robust data integrity for model training and validation.

### Biopsy and histopathological analysis

The majority of the lesions (91 out of 106) evaluated in CEM were subjected to ultrasound-guided biopsy using a 14-gauge semi-automatic needle. Core needle biopsies were performed after skin sterilization and local anesthesia, obtaining at least 3 tissue cores from the lesion. The evaluation on the remaining 15 lesions was performed using vacuum-assisted biopsy (VAB). VAB was performed using an 11-gauge semi-automatic needle under mammography guidance, following stereotactic principles with contrast agent injection to enhance lesion visibility. After a 2-minute wait, breast compression was applied, and the patient was positioned prone on the biopsy table. The optimal biopsy approach was chosen based on lesion location and breast thickness.

The lesion was localized, compressed, and imaged at multiple angles before guiding the needle with a computerized coordinate system. After antisepsis and anesthesia, the needle was advanced, and at least 12 tissue samples were obtained through multidirectional biopsies.

Finally, a stereotactic marker was placed for future localization.

Histological features, tumor and nuclear grade, along with immunohistochemical status including estrogen receptor, progesterone receptor, and HER2 status, were obtained from the final histopathological results of biopsy or surgical tumor specimens.

### CEM protocol

All CEM examinations were performed on dedicated low-dose digital mammography units that were capable of performing full-field 2D DM and CEM (Giotto Class; IMS Giotto, Bologna, Italy, for Center 1, and Senographe Essential; GE Healthcare, Chicago, IL, USA, for Center 2). Written informed consent was obtained from all patients prior to initiating the CEM examination.

Specifically, 2 minutes after the intravenous injection of 1.5 mL/kg of body weight of iodinated contrast medium (iodine concentration of 370 mgI/mL) at a rate of 2-3 mL/s, a set of images was captured in quick succession in both CC and MLO views.

Each CEM examination consisted of a low-energy (LE) acquisition (26-30 kVp), a high-energy (HE) acquisition (45-49 kVp), and the subsequent generation of dual-energy subtraction recombined images.

The details of the CEM acquisition are documented in previous studies.^12^

### Image sets

We retrospectively analyzed 106 CEM images by female patients (mean age, 58 years; range, 34-83 years) from the 2 centers (50 from Center 1 and 56 from Center 2 with the GE Healthcare system).

Specifically, dual-energy subtraction recombined images were utilized for the deep learning analysis.

Histological diagnoses classified 51 lesions (48%) as malignant and 55 lesions (52%) as benign.

A radiology resident with 3 years of breast imaging experience performed manual segmentation of the entire breast profile.

**Figure 1:**
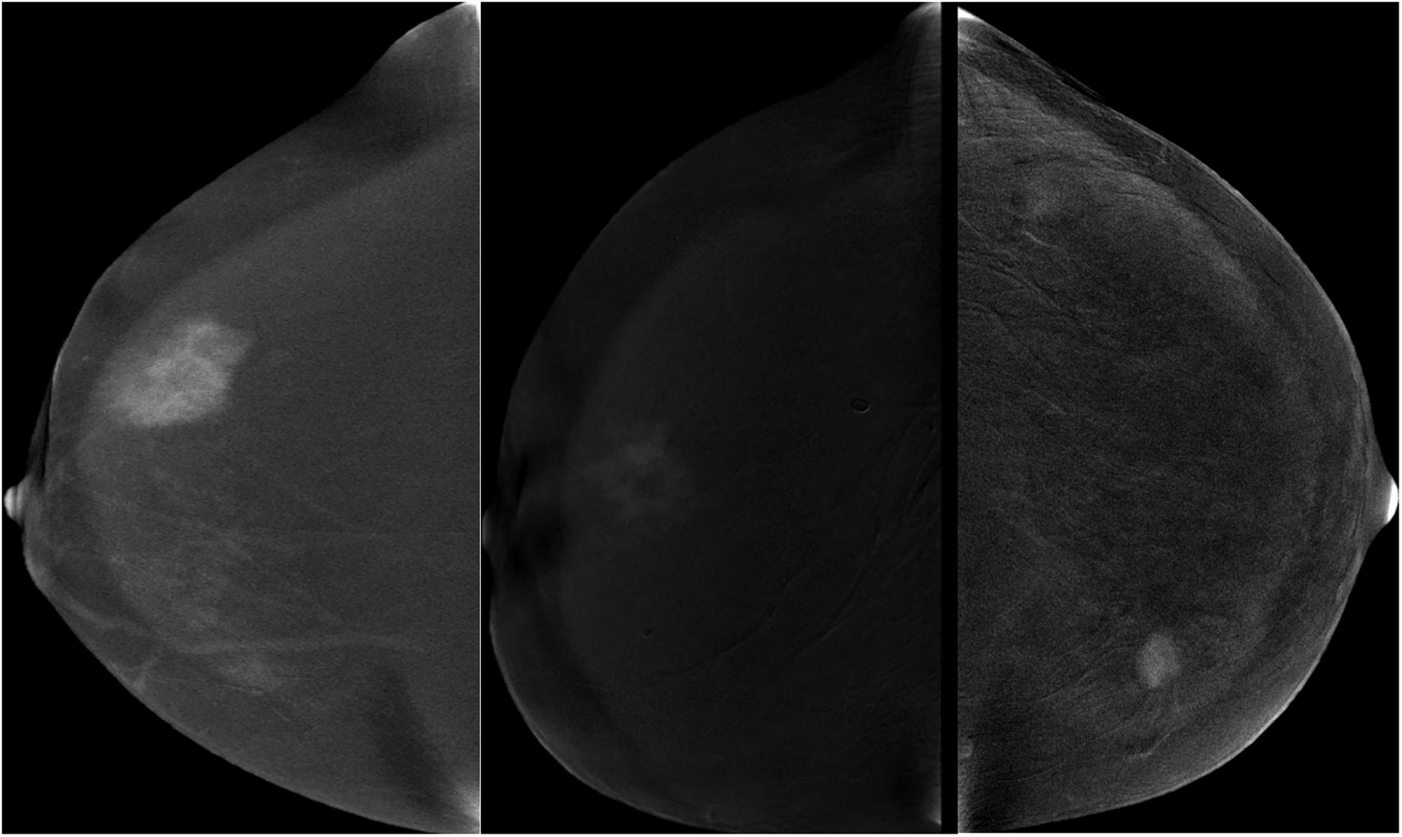
CEM images.

Those images (Figure 1) were used for the training, cross-validation, internal testing of 2 transfer learning models and external testing.

### Deep-learning modeling

The deep-learning methodology applied in this study utilizing the Trace4Research™ radiomic platform (DeepTrace Technologies S.R.L.) was used (https://www.deeptracetech.com/files/TechnicalSheet__TRACE4.pdf), which is designed to support various convolutional neural network (CNN) architectures for processing medical images. The main tasks performed included tuning, training, validating, as well as internal and external testing of the CNNs, as well as adapting medical images to fit the CNN input constraints, such as image size.

For this research, the goal was to classify images into binary categories: “malignant” or “benign.” Two prominent CNN architectures were employed: ResNet50 and DenseNet201. ResNet50 consists of 50 layers, while DenseNet201 comprises 201 layers.

Both networks are adept at learning complex features from the images.

For each CNN architecture, a 10-fold cross-validation procedure was used to train and test an ensemble of 3 CNN models. In this process, each model’s classification output was aggregated using a majority-vote rule, where the final classification was determined by averaging the class probabilities from the ensemble. To prevent data leakage, the data splitting was performed at the patient level rather than the image level.

Given the limited sample size typical of a pilot study, we adopted a transfer learning approach to mitigate the risk of overfitting. We utilized ResNet50 and DenseNet201 networks pre-trained on the ImageNet dataset; fine-tuning was then performed only on the last layers to adapt the models to the binary breast cancer classification task.

CEM images were resized to a uniform dimension of 224 × 224 pixels to match the input requirements of the CNNs.

Strictly within the training folds, automatic data augmentation techniques—such as rotation, reflection, scaling, shearing, and translation—were applied to the CEM images. Furthermore, to enhance the module’s robustness, automatic techniques were employed, particularly geometric transformations. Geometric transformations are a type of image data augmentation technique that adjust the geometric structure of images by repositioning pixels without changing their value.^16^

This augmentation enhanced image diversity and helped improve the model’s performance by providing varied training examples.

No additional data processing was applied to the CEM images beyond resizing and augmentation.

No augmentation was applied to the validation or test sets.

### Statistical analysis

Statistical analysis was conducted with embedded tools of the Trace4Research platform. To describe the training, validation, internal and external testing performance, we calculated their performance in terms of Area Under the Receiver Operating Characteristic Curve (ROC-AUC), Accuracy, Sensitivity, Specificity, Positive Predictive Value (PPV), Negative Predictive Value (NPV), together with 95% confidence interval (95% CI) and *P* value for one-sided Wilcoxon signed rank test, performed to assess statistical significance with respect to chance/random classification, with significance levels set at .05 (*) and .005 (**).

## Results

We used 106 CEM images from 2 centers and acquired with 2 different imaging systems (50 from Center 1 and acquired with Giotto Class system, and 56 from Center 2 and acquired with GE Healthcare system).

Among these, 51 lesions (48%) belonged to class “malignant” (24 and 27 from Center 1 and 2, respectively) and 55 lesions (52%) belonged to class “benign” (26 and 29 from Center 1 and 2, respectively), according to histological diagnosis from biopsy.

CEM from center 1 were used for the training, cross-validation, and internal testing.

**Figure 2:**
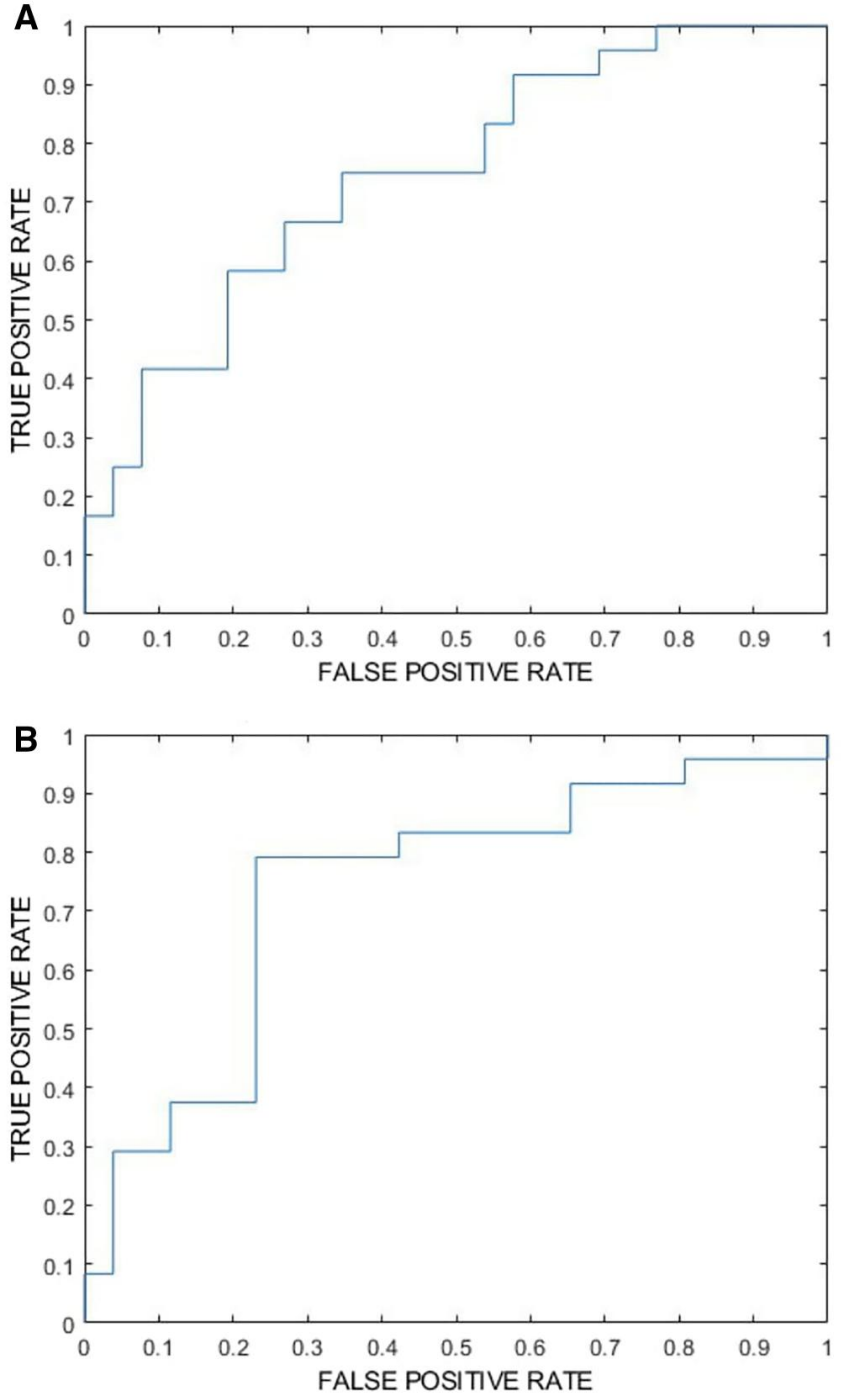
(**A**) ROC curve for the model consisting of 3 ensembles of ResNet50 architectures (from internal testing). (**B**) ROC curve for the model consisting of 3 ensembles of DenseNet201 architectures (from internal testing).

Tables 1 and 2 show ROC-AUC, Accuracy, Sensitivity, Specificity, PPV and NPV, with their 95% CIs, as obtained from the training, cross-validation, and internal testing of the 2 models consisting of 3 ensembles of deep-learning classifiers. Furthermore, for each model, the ROC curve for the 3 ensembles is plotted in Figure 2A and B.

**Table 1.** Model of 3 ensembles of ResNet50 classifiers | Classification performance in terms of AUC, Accuracy, Sensitivity, Specificity, PPV, NPV, corresponding 95% confidence interval, and statistical significance with respect to chance/random classification.

	Training	Validation	Internal testing
ROC-AUC (%) [95% CI]	99 [99-100]	77** [69-84]	72* [55-89]
Accuracy (%)[95% CI]	96**[95-97]	67**[64-70]	67** [55-78]
Sensitivity (%) [95% CI]	97 [94-100]	72** [68-76]	67** [54-79]
Specificity (%) [95% CI]	95** [92-98]	60** [52-68]	63** [47-80]
PPV (%) [95% CI]	95** [93-97]	68** [62-74]	72** [62-81]
NPV (%) [95% CI]	97** [95-100]	73** [62-84]	68** [58-77]

Performances are reported for the training, validation, and internal-testing phases.

* *P* value < 0.05/** *P* value < 0.005.

**Table 2.** Model of 3 ensembles of DenseNet201 classifiers | Classification performance in terms of AUC, Accuracy, Sensitivity, Specificity, PPV, NPV, corresponding 95% confidence interval, and statistical significance with respect to chance/random classification.

	Training	Validation	Internal testing
ROC-AUC (%) [95% CI]	100** [100-100]	79** [73-85]	75* [58-92]
Accuracy (%) [95% CI]	98** [98-99]	69** [57-80]	69** [58-79]
Sensitivity (%) [95% CI]	99** [97-100]	77 [54-100]	69** [52-86]
Specificity (%) [95% CI]	98** [97-98]	58* [33-84]	68** [65-70]
PPV (%) [95% CI]	98** [97-99]	69** [53-86]	69** [55-83]
NPV (%) [95% CI]	99** [98-100]	77** [70-84]	73** [55-92]

Performances are reported for the training, validation, and internal-testing phases.

* *P* value < 0.05/** *P* value < 0.005.

The statistical significance of the resulting classification performance with respect to chance/random classification is also reported (in terms of *P* value).

Based on ROC-AUC, the DenseNet201 ensemble model proved to be the best performing for the task of interest.

While the training set performance (AUC 100%) indicates overfitting typical of small datasets, the consistent performance between the validation (AUC 79%) and internal testing sets (AUC 75%) demonstrates the model’s stability and generalization capability on unseen data.

CEM from Center 2 were used for external testing of the best model (DenseNet201). Table 3 shows ROC-AUC, Accuracy, Sensitivity, Specificity, PPV, and NPV.

**Table 3:** Performance are reported for the external testing (accuracy, sensitivity, specificity, PPV, and NPV).

	External testing
Accuracy (%) [95% CI]	65 [51-76]
Sensitivity (%) [95% CI]	78 [59-89]
Specificity (%) [95% CI]	52 [34-69]
PPV (%) [95% CI]	60 [44-74]
NPV (%) [95% CI]	71 [50-86]

## Discussion

This study demonstrates that deep learning models based on radiomics and CEM data can assist in lesion characterization.

This deep learning model, which achieved the highest AUC value (75%), demonstrated high diagnostic sensitivity (77.8%) and a good positive predictive value (69.3%), making it a valuable tool for clinical decision support. In addition, its moderately high specificity (67.8%) and high negative predictive value (73.5%) are particularly useful for reducing unnecessary biopsies and enabling more accurate diagnostic evaluation.

These findings align with the broad range of diagnostic performance reported in a recent systematic review by Sorin et al., which identified AUC values for deep learning in CEM ranging from 0.53 to 0.99 across 16 retrospective studies.^17^ While our performance falls within this spectrum, it reflects the specific challenges of a pilot study with a limited dataset compared to larger cohorts.

We observed a performance gap between the training phase and the testing phase. The perfect scores in the training set suggest that the deep learning models, due to their high capacity relative to the sample size, memorized the training data (overfitting). However, the alignment between validation and internal testing results suggests that the overfitting was confined to the training phase and did not compromise the model’s ability to learn generalizable features for new patients.

This is a known challenge in medical image analysis when datasets are small. As noted by Kinkar et al., CEM datasets are often limited in size, making models prone to memorizing training data rather than learning generalizable features.^18^

To mitigate this, we employed data augmentation and transfer learning, techniques widely recommended to enhance robustness in small cohorts.

In this study, an external test set from another institution was used to demonstrate that the model is robust even with data coming from different centers.

This model holds the potential for enhancing early breast cancer detection rates while reducing the burden on radiologists.

Specifically, computer-aided diagnosis (CAD) based on artificial intelligence (AI) or deep learning (DL) can provide radiologists with automated analysis as a second opinion or as supportive assistance, greatly enhancing the efficiency and accuracy of their diagnostic decisions.^19^

We observed a decline in performance metrics, particularly specificity, when applying the model to the external test set (Accuracy 64.3%, Specificity 51.7%) compared to the internal validation. This drop is likely attributable to the domain shift caused by the use of different mammography systems. This performance drop is likely attributable to the domain shift caused by vendor-specific differences. As highlighted by Kinkar et al., different vendors (eg, GE Healthcare vs. Hologic vs. Siemens) employ distinct energy levels, anode/filter materials (eg, Mo/Rh vs. W/Ag), and reconstruction algorithms. These variations create significant differences in pixel intensity and texture distribution that can hinder model generalization if not harmonized.^18^

This finding underscores the importance of multi-vendor training data or image harmonization techniques in future large-scale studies.

Radiomics analysis of tumor features extracted from CEM images provides an important tool for breast cancer characterization.^13^

Despite notable heterogeneity and clear threshold effect, CEM demonstrates high diagnostic accuracy for breast cancer detection.^20^ The highest pooled sensitivity and specificity estimates were achieved when both low-energy and recombined images were analyzed, combining lesion morphology and enhancement patterns. Specifically, CEM showed a sensitivity of 95.4% when any enhancement was deemed suspicious; however, 1 in 3 cancers displayed either no enhancement or only subtle enhancement.^21^

This underscores the importance of not downgrading suspicious lesions based solely on lack of enhancement, particularly in the presence of calcifications, as ductal carcinoma in situ may not enhance.^14^

The current body of evidence on radiomics using CEM is limited, with few available studies.

Patel et al. explored the use of a CAD system with CEM to assess its diagnostic performance against that of experienced radiologists. They developed a predictive model using a support vector machine (SVM) classification technique, incorporating both morphological and textural features from low-energy and recombined images of 50 lesions. The CAD-CEM system, based on SVM, achieved a diagnostic accuracy of 90%, outperforming the individual diagnostic accuracies of the 2 radiologists, which were 78% and 86%, respectively. The study concluded that CAD-CEM can provide valuable support to radiologists, mainly by decreasing the number of false-positive results.^22^

In a separate study by the same team, Danala et al. employed a CAD approach on CEM images to classify breast masses. They utilized segmentation results from dual-energy subtracted images in a dataset of 111 breast lesions to develop a multilayer perceptron machine-learning model for mass lesion classification. This use of CAD-CEM improved the accuracy of segmenting mass regions.^23^

Comparison with recent larger studies highlights the importance of dataset scale and automation. For instance, Zheng et al. recently developed a fully automated pipeline system trained on a large multicenter cohort of 1912 patients, achieving superior performance with AUCs of 0.940 in external testing. Unlike our study, which relied on manual segmentation, their use of deep learning for automatic segmentation significantly improved workflow efficiency, taking only 6 s per lesion compared to the minutes required for manual analysis.^24^

Our study has several limitations. First, the sample size is relatively small, which is inherent to the pilot nature of this investigation and the emerging status of CEM in clinical routine. To address this, we employed data augmentation and transfer learning techniques to ensure robust model training despite the limited dataset. However, we did not incorporate prospective or international datasets.

Second, the breast profile segmentation was performed manually. Although this task carries inherently low inter-observer variability compared to lesion segmentation, it remains an operator-dependent step.

Third, the dataset has a slight imbalance between benign and malignant lesions. Although we applied a class weighting method to address this, the issue of class imbalance may not be fully resolved.

Fourth, the presence of background parenchymal enhancement may affect the model’s accuracy, particularly in the case of small lesions.

To address the suboptimal specificity observed in the external cohort (51.7%), future iterations of the model will need to incorporate clinical variables and radiological features into a multimodal fusion model. Furthermore, adjusting the classification probability threshold could help balance sensitivity and specificity according to specific clinical needs.

## Conclusions

We developed an AI-assisted diagnostic model for breast cancer showing promising feasibility. To advance toward clinical deployment, the next steps must include prospective validation on large, multivendor cohorts to ensure robustness. Ultimately, the model is envisioned to be deployed as an integrated decision-support tool, providing radiologists with a “second opinion” probability score to refine biopsy recommendations.

## Data Availability

The datasets generated and analyzed during the current study are not publicly available due to institutional restrictions and patient confidentiality policies. However, de-identified data and radiomic feature sets may be shared upon reasonable request to the corresponding author, subject to institutional approval. The code used for  feature extraction and modeling is available upon request for reproducibility purposes.
